# Circular RNA CircHIPK3 Promotes Gemcitabine Sensitivity in Bladder Cancer

**DOI:** 10.7150/jca.39722

**Published:** 2020-01-22

**Authors:** Fang Xie, Ning Zhao, Hui Zhang, Dalong Xie

**Affiliations:** 1 Medical Basic Experimental Teaching Center, China Medical University, Shenyang, 110122, China; 2 Surgery Laboratory, Affiliated First Hospital, China Medical University, Shenyang, 110001, China; 3 Department of Urinary surgery, Shengjing Hospital, China Medical University, Shenyang, 110004, China; 4 Department of Anatomy, College of Basic Medicine, China Medical University, Shenyang, 110122, China

**Keywords:** bladder cancer, circular RNA, circHIPK3, chemotherapy, gemcitabine

## Abstract

**Purpose**: Recent studies showed circular RNA (circRNA) played important regulatory roles in tumors, including genesis of chemotherapy resistance. In this study, the role of circHIPK3 on chemotherapy resistance of bladder cancer (BC) will be clarified.

**Methods**: Real-time quantitative PCR was applied to examine the circHIPK3 expression. The gemcitabine sensitivity and cell proliferation viability were analyzed by Cell Counting Kit-8 assay. Double-stained flow cytometry was used to detect the cell apoptosis.

**Results**: In BC tissues and cell lines, the circHIPK3 expression was down-regulated. Its expression had a negative correlation with pathological grade, lymph node metastasis and gemcitabine insensitivity of BC patients. CircHIPK3 was a independent prognostic biomarker for BC patients. The expression of circHIPK3 in T24/gem and J82/gem cell lines (resistant to gemcitabine) was down-regulated significantly. The over-expression of circHIPK3 decreased IC50 of gemcitabine and promoted gemcitabine's cytotoxicity in T24/gem and J82/gem cells.

**Conclusions**: The circHIPK3 is low-expressed in BC and is an independent prognostic biomarker for BC patients. The low-expression of circHIPK3 is associated with the insensitivity to gemcitabine of BC patients, over-expression of circHIPK3 promotes gemcitabine sensitivity in BC.

## Introduction

Bladder cancer (BC) is one of the ten most common tumors in the whole body and the most common malignant tumors in urinary system 1. In the West, the incidence of BC is second only to prostate cancer in urogenital tumors, while it occupies the first place in China 1-3. Bladder urothelial carcinoma is the most common pathological type of BC, accounting for more than 90% of the total number of BC patients.

Chemotherapy is one of the most effective means to treat BC at present, which reduced significantly the risk of recurrence and metastasis, and improve evidently the prognosis of BC patients 4. Nevertheless, the resistance of cancer cells to chemotherapy drugs often leads to chemotherapy failure.

Recently, circular RNA (circRNA) is a hotspot in the field of life science research, it is a special kind of non-coding RNA molecule. Because the closed ring structure is not affected by RNA exonuclease, circRNAs are more stable and difficult to degrade. CircRNA molecules are rich in microRNA binding sites and act as competitive endogenous RNA (ceRNA) to play the role of microRNA sponge in cells 5,6. By interacting with tumor-associated microRNAs, circRNAs play important regulatory roles in tumorigenesis 7-10.

CircHIPK3 (circRNA ID: hsa_circ_0000284), also named bladder cancer-related circular RNA-2 (BCRC-2), originates from the second exon of Homeodomain-interacting protein kinase 3 (HIPK3) gene and consists of 1099 nucleotides in length. Recent studies confirmed that circHIPK3 showed the abnormal expression level and functions in various tumors, included prostate cancer, gastric cancer, lung cancer, ovarian cancer, and so on 11-16. In addition, circHIPK3 contributed to lung fibroblast-to-myofibroblast transition in pulmonary fibrosis 17, and promoted the proliferation and migration of cardiac fibroblasts in cardiac fibrosis 18.

CircHIPK3 was low-expressed in BC, up-regulation of circHIPK3 suppressed the angiogenesis, invasion, and migration of BC cells, and inhibited the growth and metastasis of BC in vivo 19. In our preliminary experiment, circRNA microarray screening found that circHIPK3 was also down-regulated in BC tissues, which suggested circHIPK3 might be involved in the genesis of BC. Nevertheless, it is not clear whether circHIPK3 is involved in the formation of chemotherapy resistance in BC and its role in chemotherapy resistance.

## Methods

### Clinic specimens

68 BC tissue and corresponding normal bladder urothelial tissue (NBUT) specimens were obtained Shengjing Hospital from August 2012 to April 2014. All tissue specimens were collected through cystoscopy and confirmed by pathological diagnosis. All patients received no chemotherapy, radiotherapy or other antineoplastic treatment before diagnosis. The inclusion criteria of patients was that all patients were diagnosed as BC, and they needed neoadjuvant chemotherapy with gemcitabine before operation or they chose gemcitabine chemotherapy if they could not be treated surgically. The exclusion criteria was the BC patient did not treat with gemcitabine chemotherapy.

After definite diagnosis, all patients received gemcitabine treatment. The curative effects are verified according to cystoscopy and enhancement CT scanning after three cycles of chemotherapy. All patients were divided into sensitive group (n = 38) and insensitive group (n = 30) according to the therapeutic effects.

### Cell lines and culture

Human fetal bladder tissue derived CCC-HB-2 cell line, human bladder epithelial immortalized cell line SV-HUC-1 and BC cell lines (T24, J82, UMUC3) were stored in our laboratory and identified by STR Authentication. T24/gem and J82/gem cell lines (resistant to gemcitabine) were established in previous studies 20,21. All cells were cultured in DMEM medium with 10% fetal bovine serum (ExCell Bio, Shanghai, USA) in a 37°C incubator.

### Real-time quantitative PCR

The extraction, purification and identification of RNA were applied all according to our previous studies 20,21. The A260/A280 ratio of purified RNA was typically between 1.8 and 2.4 and the yield between 80µg and 120µg. RNA samples were stored at -80°C. RNA integrity was assessed by gel electrophoresis. RNase R was used to eliminate the linear RNAs. One Step SYBR RT-PCR Kit (Takara, Dalian, Liaoning, China) was used to qualify the expression level of circHIPK3 according to manufacturer's instructions using a 7700 PCR System (Applied Biosystems, ThermoFisher, Foster City, CA, USA). Each reaction contained 2×One Step SYBR RT-PCR Buffer (12.5 µL), PrimeScript 1 Step Enzyme Mix 2 (1 µL), Forward Primer (10µM, 1 µL), Reverse Primer (10µM, 1.25 µL), RNA template (2 µL) and RNase Free dH2O (7.5 µL). The cycling condition is as follows: Stage 1: 42℃ for 5 min, 95℃ for 10 sec,1 Cycle; Stage 2: 95℃ for 5 sec, 60℃ for 30 sec, 40 Cycles; Stage 3: 95℃ for 15 sec, 60℃ for 30 sec, 95℃ for 15 sec, 1 Cycles. The primers for circHIPK3 were 5'- TTCAACATATCTACAATCTCGGT -3' (sense) and 5'- ACCATTCACATAGGTCCGT -3' (antisense) 19. After normalization with reference to expression of GAPDH, the relative expression level of circHIPK3 was calculated as 2^-ΔΔCT^ method.

### Cells Transfection

The expression plasmid and negative control plasmid of circHIPK3 (pCD5-circHIPK3 and pCD5-NC) were constructed by GENESEED Company (Guangzhou, Guangdong, China). Lipofectamine™ 3000 (Thermo Fisher Scientific, USA) was applied to transfect plasmids into cells according to manufacturer's instructions.

### Cell viability detection

Cell Counting Kit-8 (MedChemExpress, Monmouth Junction, NJ, USA) was used to examine cell viability. 5×10^3^ cells were suspended with 100 µl culture medium and seed in a well of a 96-well plate; 10 μl CCK-8 solution was added to each well of the plate; the plate was incubated 1 h in the incubator and mix gently on an orbital shaker for 1min. The absorbance at 450 nm was measured using a microplate reader.

### Gemcitabine sensitivity detection

5×10^3^ cells were suspended with 100 µl culture medium and seed in a well of a 96-well plate; new culture medium with gemcitabine (0.1 μg/ml, 0.5 μg/ml, 1 μg/ml, 5 μg/ml and 10 μg/ml respectively) (Sigma-Aldrich, St. Louis, MO, USA) was added to replace old culture medium 6 h later; after 24 h, cell viability was examined and the half maximal inhibitory concentration (IC50) was calculated according to our previous studies 20,21.

### Cell apoptosis detection

Annexin Ⅴ-FITC apoptosis detection kit (Meilunbio, Dalian, Liaoning, China) was applied to detect cell apoptosis rate. 1×10^5^ cells were suspend in 100 µl culture medium and added 5 µl Annexin V-FITC and 5 µl PI; cell suspension incubated at room temperature and without light for 15 min; 400 µl Binding Buffer was added and and mixed gently; then cell apoptosis rate was examined and analyzed according to our previous studies 20,21.

### Statistical analysis

All data was analyzed using Graphpad prism 5 (GraphPad Software, San Diego, CA, USA). All BC patients were divided into two groups (high-expression and low-expression) according to the median value of relative circHIPK3 expression. The difference comparison was analyzed by one-way ANOVA, Student's t-test and Chi-square test. The survival rate was calculated with Kaplan-Meier method with the log-rank test for comparisons. Variables with a value of *P*<0.05 in the univariate analysis were included in the subsequent multivariate analysis based on the Cox proportional hazards model. A *P* value of less than 0.05 was considered to be statistically significant.

## Results

### The expression of circHIPK3 was down-regulated in BC

**Figure 1:**
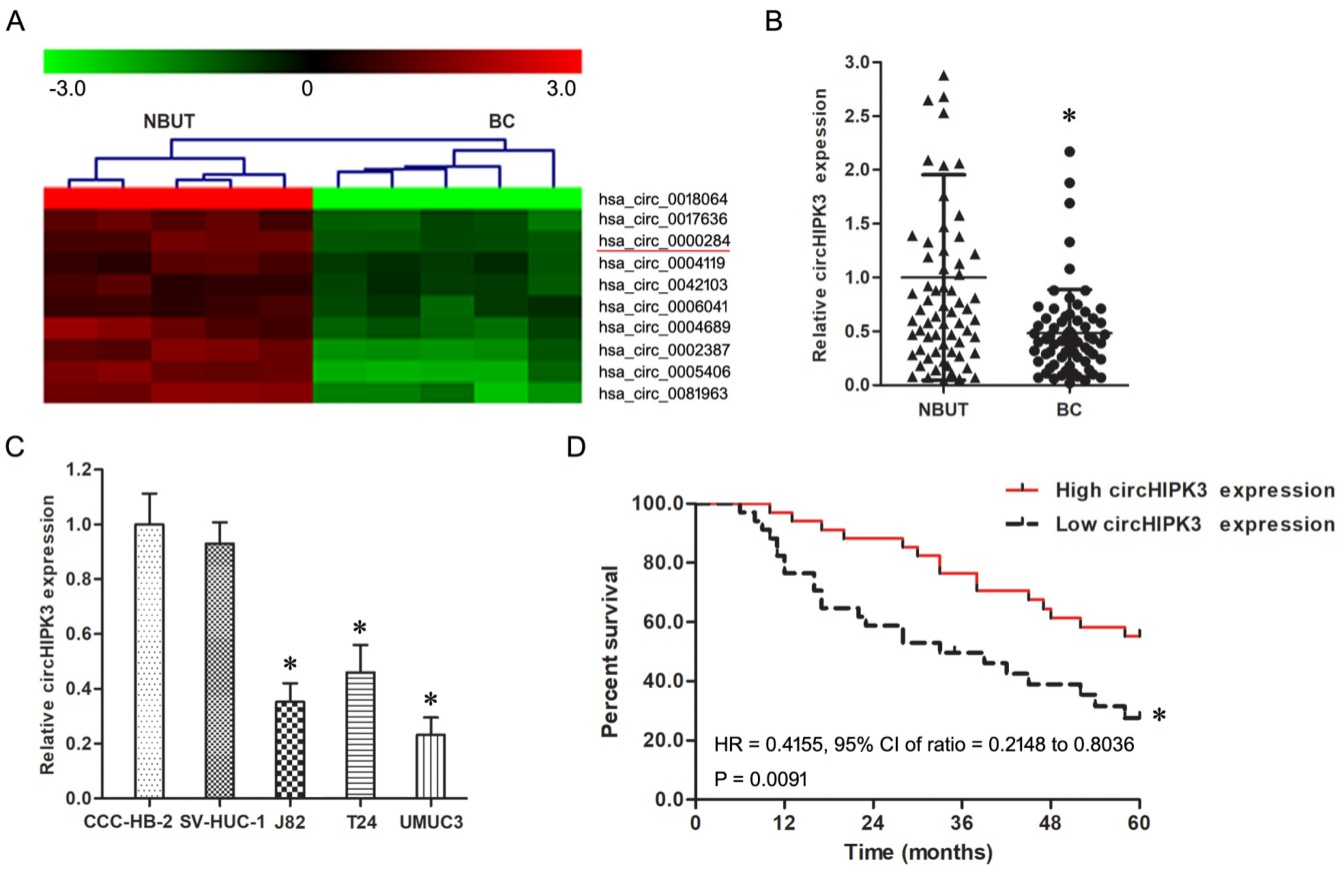
** The expression of circHIPK3 was down-regulated in BC. A:** Representative down-regulated circRNAs in BC compared with NBUT analyzed by microarray assay (n=5). **B:** The expression of circHIPK3 in BC was down-regulated compared with NBUT (n=68). * *P*<0.05 vs. NBUT. **C:** The expression of circHIPK3 in T24, J82 and UMUC3 cells was down-regulated in comparison with CCC-HB-2 and SV-HUC-1 cells. * *P*<0.05 vs. CCC-HB-2 and SV-HUC-1 cells. **D:** Kaplan-Meier analysis indicated that BC patients with lower circHIPK3 expression exhibited a shorter survival time than those with higher expression. * *P*<0.05 vs. Low circHIPK3 expression group.

Firstly, the circRNAs which differentially expressed between BC and NBUT were screened using circRNA microarray assay, screening results suggest that the expression of circHIPK3 had a prominent down-regulation in BC compared with NBUT (Figure 1A). Secondly, real-time quantitative PCR confirmed circHIPK3 was significantly low-expressed in BC in comparison to NBUT (Figure 1B, *P*<0.05). Thirdly, the circHIPK3 expression in T24, J82 and UMUC3 cells was much lower than that in CCC-HB-2 and SV-HUC-1 cells (Figure 1C, *P*<0.05).

As shown in Table 1, the circHIPK3 expression had a negative correlation with high pathological grade, lymph node metastasis and gemcitabine chemotherapy insensitivity of BC patients (*P*<0.05), but was not correlated with age, gender, smoking history and muscular invasion of BC patients (*P*>0.05).

### circHIPK3 was an independent prognostic biomarker for BC patients

The 5 year disease free survival rate (DFS) in lower or higher circHIPK3 expression group were 32.3% and 55.9% respectively, the Kaplan-Meier analysis found BC patients with lower circHIPK3 expression exhibited a shorter survival time than those with higher circHIPK3 expression (Figure 1D, *P*<0.05). Moreover, Cox regression analysis showed that lower circHIPK3 expression was an independent prognostic biomarker for DPS in BC patients (Table 2).

### Low-expression of circHIPK3 correlated to gemcitabine resistance in BC

In consideration of the negative correlation between the circHIPK3 expression and gemcitabine insensitivity of BC chemotherapy, the circHIPK3 might participate in the chemotherapy resistance of BC. In order to test this hypothesis, the circHIPK3 expression was examined in gemcitabine-resistant cell lines.

**Figure 2:**
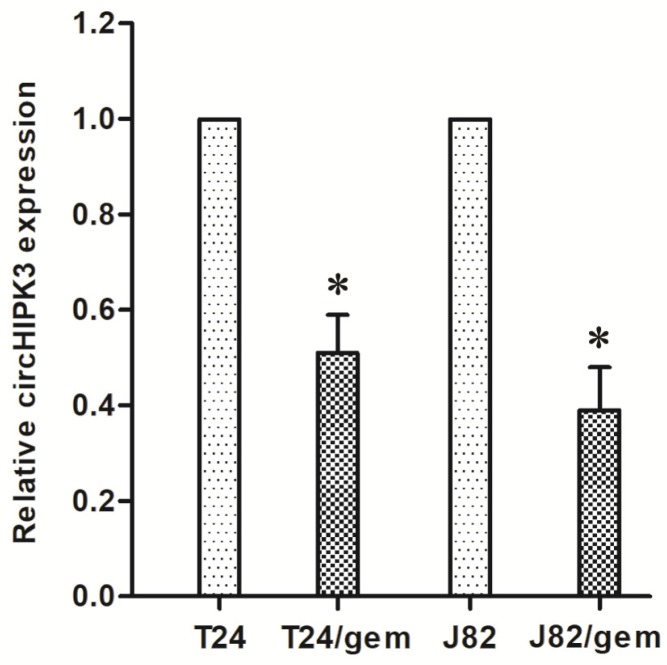
Compared with T24 and J82 cells, the expression of circHIPK3 in gemcitabine-resistant T24/gem and J82/gem cell lines was significantly down-regulated. * *P*<0.05 vs. T24 and J82 cells.

The results showed the circHIPK3 expression in T24/gem and J82/gem cell lines was significantly down-regulated as compared to their parental cells in vitro (Figure 2, *P*<0.05). This data suggested that low-expression of circHIPK3 correlated to gemcitabine resistance in BC.

### Over-expression of circHIPK3 re-sensitized gemcitabine-resistant BC cells to gemcitabine

**Figure 3:**
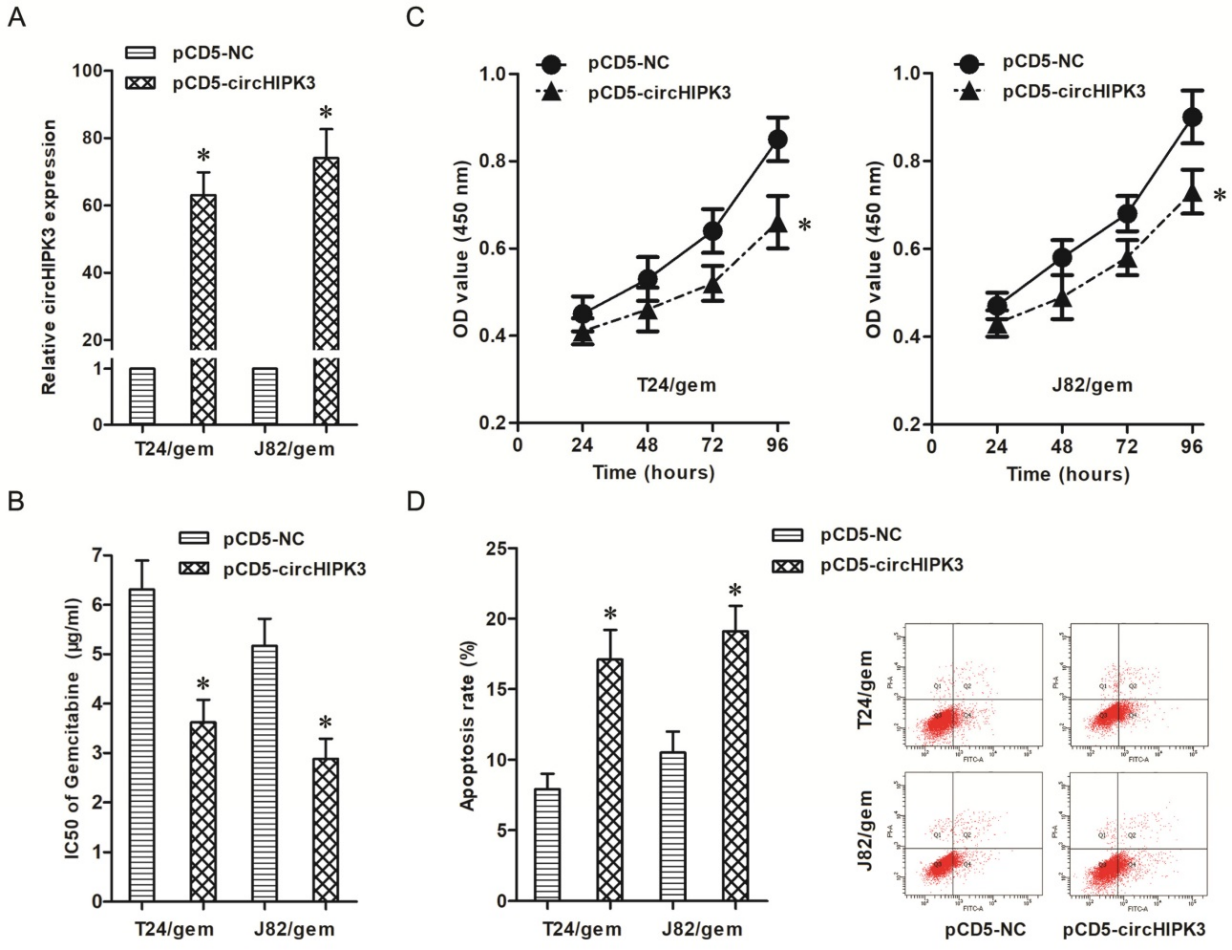
** Up-regulation of circHIPK3 re-sensitized gemcitabine-resistant BC cells to gemcitabine. A:** Transfection of pCD5-circHIPK3 up-regulated the expression of circHIPK3 in T24/gem and J82/gem cells. **B:** Over-expression of circHIPK3 decreased the IC50 of gemcitabine in T24/Gem and J82/gem cells **C:** Over-expression of circHIPK3 suppressed cell proliferation in T24/Gem and J82/gem cells treated with 0.5 μg/ml Gemcitabine. **D:** Over-expression of circHIPK3 promoted apoptosis in T24/Gem and J82/gem cells treating with 0.5 μg/ml Gemcitabine. * *P*<0.05 vs. pCD5-NC group.

To verify the roles of circHIPK3 on chemotherapy resistance, pCD5-circHIPK3 transfected into T24/gem and J82/gem cells to up-regulated the circHIPK3 expression (Figure 3A, *P*<0.05). And, over-expression of circHIPK3 decreased the IC50 of gemcitabine in T24/gem and J82/gem cells (Figure 3B, *P*<0.05), which certified that over-expression of circHIPK3 re-sensitized T24/gem and J82/gem cells to gemcitabine.

In addition, over-expression of circHIPK3 inhibited cell viability and advanced cell apoptosis (Figure 3C & 3D, *P*<0.05) in T24/gem and J82/gem cells treated with 0.5 μg/ml gemcitabine. Over-expression of circHIPK3 significantly promoted gemcitabine's cytotoxicity in gemcitabine-resistant BC cells.

## Discussion

Increasing researches found some circRNAs was involved in the genesis and progress of BC. For instance, circMTO1 was down-regulated in BC, its expression level was negatively correlated with metastasis and poorer survival of BC patients 22. Lu Q et al. reported that circSLC8A1 was low-expressed in BC, circSLC8A1 up-regulation suppressed proliferation, migration and invasion of BC cells 23. Circ-0000285 was down-regulated significantly in BC tissues and serum, its expression as was associated with TNM stage, differentiation, tumor size, lymph node metastasis and distant metastasis, and the circ-0000285 expression was lower in cisplatin-resistant BC patients than in those who were cisplatin-sensitive, which suggested that circ-0000285 was an independent prognostic factor for the outcome of BC patient 24.

Firstly, real-time quantitative PCR and microarray assays found circHIPK3 was low-expressed in BC tissues and cell lines, and its expression was negatively related with pathological grade and lymph node metastasis of BC. Moreover, circHIPK3 was a independent prognostic biomarker for BC patients, patients with lower circHIPK3 expression showed a significantly worse prognosis. Thus, circHIPK3 played important roles in the genesis and progress of BC.

Li Y et al. reported circHIPK3 was low-expressed in BC, and negatively correlated with grades, invasion and lymph node metastasis of BC patients, which was consistent with our findings and further demonstrated the roles of circHIPK3 in the genesis and progression of BC 22. However, circHIPK3 was found to be highly expressed in glioma and elevated level of circHIPK3 was linked to poor prognosis 25. This suggested that our circHIPK3 played different roles in different malignant tumors.

In addition, the low-expression of circHIPK3 was related with insensitivity of BC patients to gemcitabine, which suggested circHIPK3 might be involved in chemotherapy resistance of BC. Furtherly, circHIPK3 was down-regulated in gemcitabine-resistant T24/gem and J82/gem cells compared with their parental cells. These findings preliminarily confirmed circHIPK3 took part in the genesis of chemotherapy resistance in BC.

Therefore, the impacts of circHIPK3 on gemcitabine sensitivity in BC were examined through a series of gain of function assays. The results showed over-expression of circHIPK3 reduced IC50 of gemcitabine and advanced gemcitabine's cytotoxicity in gemcitabine-resistant cells, which showed over-expression of circHIPK3 re-sensitized gemcitabine-resistant BC cells to gemcitabine. However, the underlying mechanism is unclear.

It is well known that some circRNA molecules are rich in microRNA binding sites and can act as ceRNAs to adsorb microRNAs. For example, circAKT3 could up-regulate PIK3R1 through adsorbing miR-198, and then enhance cisplatin resistance in gastric cancer 26; circPAN3 sponge miR-153-5p/miR-183-5p to mediate the doxorubicin chemoresistance in acute myeloid leukemia 27. Recently, three literatures reported circHIPK3 could sponge miR-124 in lung cancer, gallbladder cancer and hepatocellular carcinoma 28-30. In addition, miR-124 regulated the gemcitabine resistance in pancreatic cancer and enhanced gemcitabine-induced cell apoptosis 31. These findings give us a nice enlightenment, accordingly, we hypothesize circHIPK3 act as ceRNA to sponges some microRNAs and then regulate their target genes, and this mechanism is involved in genesis of chemotherapy resistance in BC.

In conclusion, the circHIPK3 is low-expressed in BC and is an independent prognostic biomarker for BC patients. The low-expression of circHIPK3 is related with the gemcitabine insensitivity of BC patients, over-expression of circHIPK3 promotes gemcitabine sensitivity in BC. Our study helps to expound the mechanism of chemotherapy resistance in BC, and future study may provide a novel therapeutic target for BC. However, whether our hypothesis is correct or whether there are other possible regulatory mechanisms has not been studied in this study, which needs to be further explored.

## Figures and Tables

**Table 1:** The association of the circHIPK3 expression and clinical pathological factors of 68 BC patients

Factors			Relative circHIPK3 expression		
		Number	High	Low	*P* value
Age	< 55	34	15	19	0.332
	≥ 55	34	19	15	
Gender	Male	39	21	18	0.462
	Female	29	13	16	
Smoking history (more than ten years)	No	42	24	18	0.134
	Yes	26	10	16	
Grade	Low grade	36	23	13	0.015*
	High grade	32	11	21	
Muscular invasion	No	38	24	14	0.052
	Yes	30	10	20	
Lymph node metastasis	No	43	26	17	0.024*
	Yes	25	8	17	
Gemcitabine chemotherapy	Sensitive	38	25	13	0.003*
	Insensitive	30	9	21	

Chi-square test, * *P*<0.05

**Table 2:** The influence of the circHIPK3 expression and clinical characteristics on DFS in BC patients

Factors	Univariate analysis			Multivariate analysis		
	HR	95% CI	*P* value	HR	95% CI	*P* value
The circhipk3 expression	4.315	1.597-8.221	0.006*	3.364	1.297-8.768	0.007*
Grade	2.202	1.035-2.395	0.043			
Lymph node metastasis	0.276	0.035-1.288	0.076			

* *P*<0.05
